# Artificial Intelligence in Personalized Medicine for Diabetes Mellitus: A Narrative Review

**DOI:** 10.7759/cureus.91520

**Published:** 2025-09-03

**Authors:** Kaushik Ghosh, Sudip Chandra, Sonali Ghosh, Uday S Ghosh

**Affiliations:** 1 Medicine, Murshidabad Medical College and Hospital, Berhampore, IND; 2 Biostatistics, Social and Official Statistics Unit, Indian Statistical Institute, Kolkata, IND; 3 Emergency Medicine and Critical Care, Seth Sukhlal Karnani Memorial Hospital, Institute of Post Graduate Medical Education and Research, Kolkata, IND; 4 Medicine, Barasat Government Medical College and Hospital, Barasat, IND

**Keywords:** ai tools, diabetes mellitus, glycemic fluctuations, indian phenotype, personalized medicine (pm)

## Abstract

Diabetes mellitus (DM) is a long-term metabolic condition involving persistent hyperglycemia, which causes morbidity, mortality, and economic stress. This article examines the role of artificial intelligence (AI)-initiated precision medicine in optimizing type 2 diabetes mellitus management in the Indian population. An exhaustive review of AI-based platforms used for diabetic treatment was performed, highlighting the combination of multidimensional data sets involving genetic, epigenetic, phenotypic, and environmental variables. The envisioned AI platform aims to offer personalized glycemic forecasts, tailored therapeutic interventions, complication monitoring, and stage-by-stage disease progression predictions. Precision medicine enabled by AI has shown promising outcomes in improving diabetes management through the administration of patient-specific treatment regimens, early glycemic change detection, and real-time monitoring of diabetes-related complications. The use of AI applications enables patients to follow evidence-based self-management behaviors, such as diet modifications, physical activity changes, insulin management, and continuous glucose monitoring. This patient-centered strategy enhances clinical efficacy, prevents long-term complications, and lowers healthcare costs. Additional longitudinal and multicentric trials are needed to confirm outcomes among heterogeneous cohorts and to fine-tune AI algorithms for increased clinical relevance and translational use.

## Introduction and background

Diabetes mellitus, a metabolic disease of chronic nature with a worldwide prevalence that is growing rapidly, carries a high health and economic cost, and new management approaches are urgently needed. The growing burden highlights the need for the development of novel solutions, including artificial intelligence (AI) enabled precision medicine, which tailors interventions to patient-specific needs. AI refers to computer programs that are used to execute tasks that would otherwise require human intelligence to accomplish, such as learning, reasoning, and problem-solving [1]. AI operates in medical decision-making by scanning vast databases using processes such as pattern recognition, which identifies trends in patient symptoms or scans, and data integration, which aggregates multiple sources, such as electronic health records, genetic information, and data from wearable devices, to make predictions or suggestions [2]. For instance, AI software can detect early signs of disease, such as diabetes, by recognizing subtle correlations that human clinicians may not observe [3]. In comparison to clinician manual inspection and rule-based methodology, AI is better suited to handling large volumes of information at high speed, with the ability to deliver scalable, personalized analysis while minimizing human error. However, AI is a complement to human knowledge, requiring calibration to provide accountability and responsible use. This makes AI particularly groundbreaking for uses, such as tailored medicine, facilitating improved and enhanced care for patients who are not literate in the technology [4]. Continuous glucose monitoring (CGM), a device that monitors blood glucose in real-time, is used to gauge hyperglycemia, a characteristic of diabetes mellitus, which has increased in prevalence by a factor of three over the last 20 years [5]. Maintaining blood glucose levels within a therapeutically acceptable range requires lifelong self-management, as deviations can cause hypoglycemia-induced coma or hyperglycemia-related microvascular and macrovascular damage. Precise prediction of blood glucose levels and insulin action delays, influenced by daily activities, lifestyle, and patient-specific traits such as body mass index (BMI), environmental disruptions (including stress and disease), and insulin sensitivity, helps reduce adverse glycemic events [6,7].

Precision medicine, which customizes care based on individual patient characteristics, improves outcomes and reduces costs by leveraging comprehensive data [8,9]. It integrates genetic, lifestyle, and environmental data to create personalized treatment plans, particularly effective for complex conditions like diabetes [10]. AI tools, including CGM-linked smartphone apps, deep learning algorithms for insulin dosing, and predictive models for disease progression, customize therapies for the Indian T2D phenotype, characterized by high insulin resistance and abdominal adiposity. Deterministic algorithms or computations involving clinical and biochemical markers and family history have been devised to identify individuals with prospects for next-generation sequencing testing for monogenic diabetes [11,12]. Precision diagnosis combines clinical characteristics, epidemiological information (sex, age, and ancestry at diagnosis), and diagnostic laboratory findings (Type 1 Diabetes-Genetic Risk Score {T1D-GRS}, type of autoantibodies, basal, and/or stimulated c-peptide measurement) to establish subdivisions for patients with type 1 diabetes (T1D), which accounts for approximately 10% of the diabetic population [13]. These clinical characteristics (sex, age, ethnicity, BMI, HbA1c, and homeostatic model) help identify individuals who respond favorably, poorly, or adversely to a group of anti-diabetic drugs, such as sulfonylureas, dipeptidyl peptidase 4 inhibitors (DPP4i), and thiazolidinediones [14,15]. Twelve classes of diabetes medication have been discovered for type 2 diabetes (T2D) [16].

**Figure 1:**
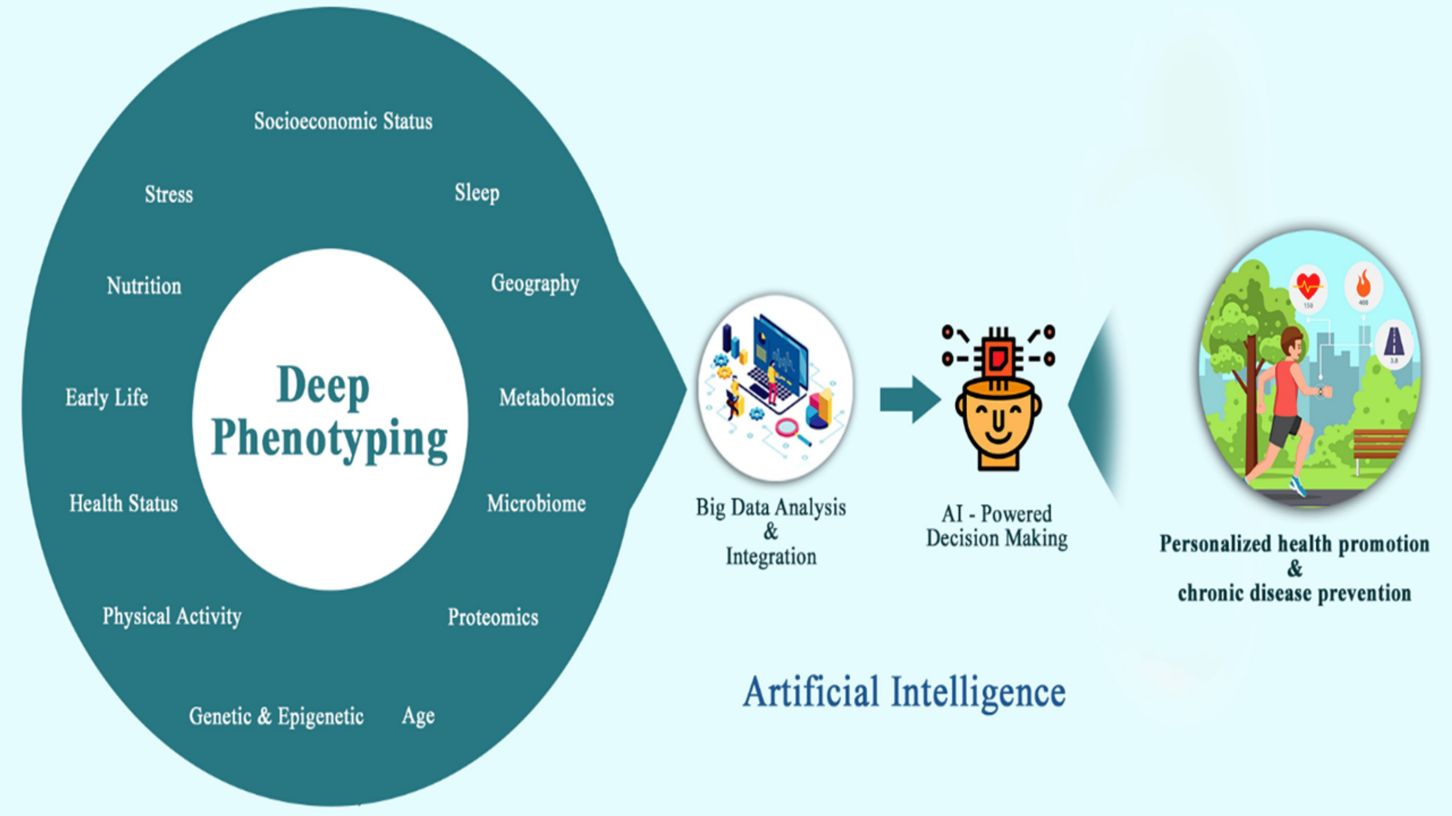
Use of AI in disease management and personalized health promotion. This figure is created by the author(s) of this study.

With the use of comprehensive clinical and biological datasets, artificial intelligence (AI) can forecast risks associated with diseases more accurately. AI-driven precision medicine gives physicians the ability to individually customize early therapies for each patient (Figure 1). AI tools analyze large datasets to predict disease risks and tailor interventions, enhancing diabetes management, especially for populations like the Indian T2D phenotype, which has unique genetic and lifestyle factors. Several AI-based tools, including CGM devices that are linked to smartphone apps and deep learning algorithms that acquire insulin infusion profiles and blood glucose levels, have been developed to address the problem of differentiating treatments based on population variability [17].

This narrative review integrates peer-reviewed evidence and experts' views on AI-based precision medicine in T2D for the Indian phenotype. It discusses AI-based tools for tailored glycemic prediction, individualized therapeutic interventions, complication monitoring, and prediction of stage-related disease progression. It aimed to improve patient-specific treatment protocols and facilitate real-time monitoring. It calls for additional multicentric and longitudinal studies to cross-validate AI algorithms in various populations, enhance clinical results, decrease long-term complications, and minimize healthcare costs. This review discusses various AI techniques applied to diabetes monitoring, focusing on aspects such as continuous glucose monitoring (CGM) integration, complication detection (including retinopathy, neuropathy, and foot ulcers), hypoglycemia prediction, disease progression tracking, and personalized blood glucose forecasting. These techniques are integrated with CGM devices and metrics, such as time in range (TIR), glucose management index (GMI), and glucose variability (GV), to enable proactive, patient-centered monitoring.

## Review

Methodology

This narrative review synthesizes existing literature on the application of AI in personalized medicine for T2D, with a focus on the Indian phenotype. A comprehensive literature search was conducted across electronic databases including PubMed, Scopus, Google Scholar, and Web of Science, using keywords such as "artificial intelligence," "machine learning," "diabetes mellitus," "personalized medicine," "continuous glucose monitoring," "Indian phenotype," and combinations thereof, covering publications from January 2010 to August 2025. The inclusion criteria encompassed peer-reviewed articles, reviews, clinical studies, and expert commentaries in English that addressed AI tools for diabetes prediction, monitoring, complication detection, and management, particularly those relevant to T2D characteristics, such as insulin resistance and abdominal adiposity in South Asian populations. Exclusion criteria involved non-English publications, studies unrelated to AI or diabetes, and those lacking empirical data or focusing solely on type 1 diabetes without overlap to T2D.

Indian phenotype characteristics among patients with type 2 diabetes mellitus

**Figure 2:**
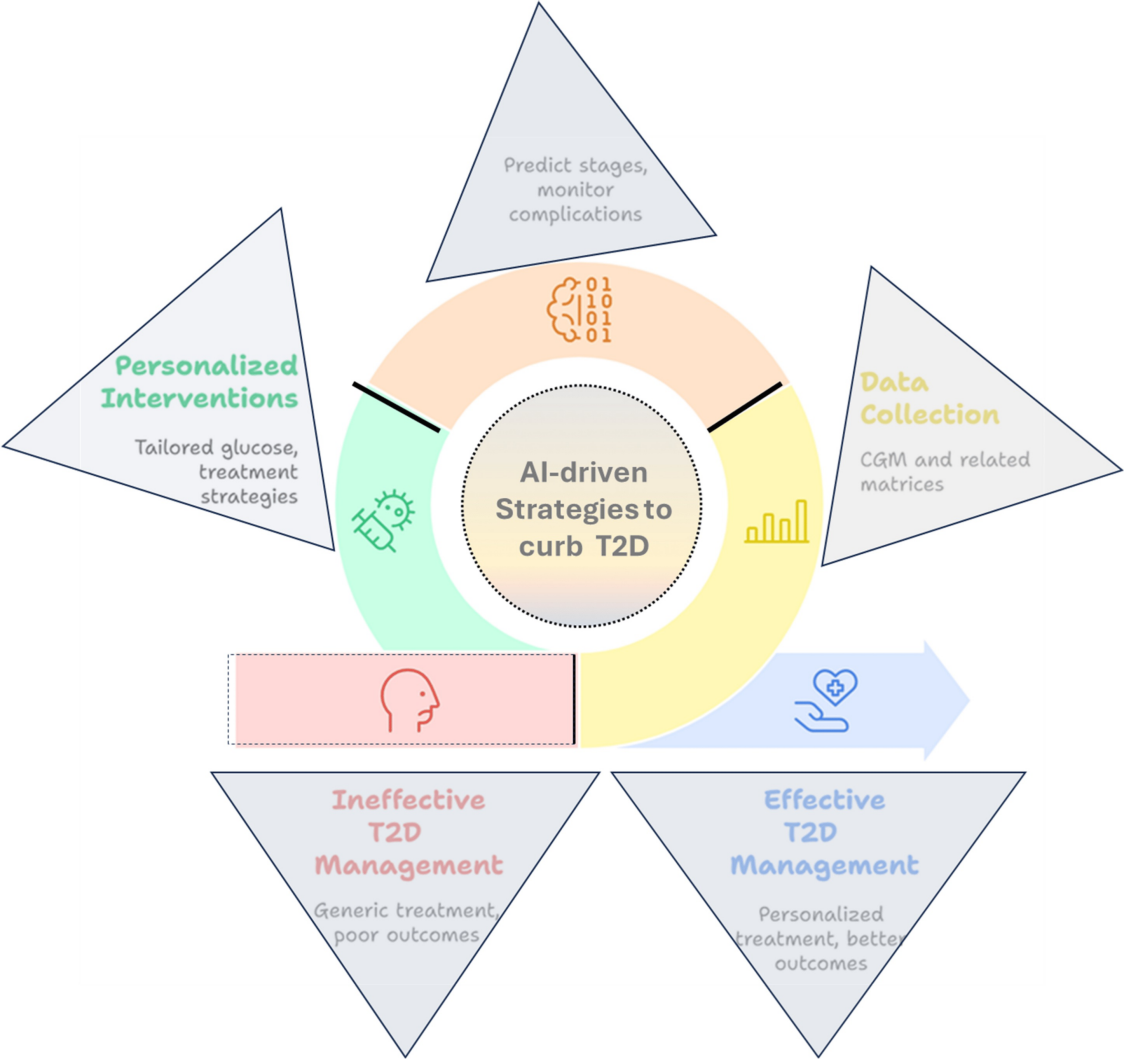
Overview of AI-driven stages for the detection of T2D in the Indian population. CGM: continuous glucose monitoring This figure is created by the author(s) of this study.

The Asian Indian phenotype is characterized by abdominal adiposity, high insulin resistance, despite lower BMI, elevated high-sensitivity C-reactive protein, low adiponectin, and increased T2D risk. The Yajnik-Yudnik (Y-Y) paradox highlights higher body fat percentages in South Asians despite lower BMI. Dyslipidemia, characterized by elevated triglycerides, inadequate high-density lipoprotein (HDL), and lower low-density lipoprotein (LDL), increases the risk. Body fat percentage (BF%) assessment can aid in early prevention. Additional research is needed to investigate T2D variation across Indian states, given the limited sample sizes in the current study [16-19]. Figure 2 represents the various stages utilizing AI highlighted for monitoring T2D in the Indian population.

A cluster-based analysis of individuals with diabetes mellitus (less than five years duration) using GAD antibodies in an Indian cohort from nine states revealed a higher prevalence of severe insulin-deficient diabetes (26.2% versus 17.5%), younger age at onset, lower BMI, and reduced β-cell function compared to the Swedish All New Diabetics in Scania (ANDIS) cohort. The findings were consistent when tested across a larger sample from 15 Indian states, despite potential selection bias [20].

Continuous glucose monitoring (CGM) devices and matrices

Continuous glucose monitoring (CGM) devices can potentially upload glucose data to electronic medical records (EMR), computers, or cloud systems, allowing for seamless monitoring and management. Every 1-15 minutes, CGM systems can broadcast glucose levels to various devices, such as watches, phones, insulin pumps, or receivers. In this section, different CGM matrices and their impact have been described.

CGM data sufficiency

Data sufficiency in CGM refers to the adequacy of data collection duration to reliably represent glucose patterns for diabetes management. The mean glucose, duration in range, and hyperglycemia measurements show strong correlations between 14 days and three months' worth of CGM data, according to a recent study [21]. The likelihood that the data are a trustworthy representation of typical patterns increases when at least 70% or roughly 10 days of CGM wear are recorded during those 14 days. There was no correlation between average glucose levels with glycemic variability or hypoglycemia. It does have a strong correlation with A1c and hyperglycemia measurements. When used alone, it offers little understanding of glucose patterns.

Glucose management index (GMI)

Similar to how HbA1c offers an average glucose level over the previous two to three months, the glucose management index (GMI) is a statistic derived from CGM data that calculates the average blood glucose level over time. A CGM device's average glucose level is used to generate GMI, which gives an estimate of the HbA1c level that would result from maintaining the current average glucose level. Using a particular formula, GMI is computed using the average glucose reading from a CGM device [22,23]. The standard formula that is applied is as follows:

GMI (%) = 3.31 + 0.02392 × average glucose (mg/dL)

For example, if the average glucose level recorded by a CGM device is 150 mg/dL, the GMI would be:

GMI (%) = 3.31 + 0.02392 ×150 mg/dL ≈ 6.898%

This implies that the estimated HbA1c would be around 6.9% if the average glucose value is kept steady. Thus, GMI is a very important instrument in diabetes care since it offers immediate feedback on glucose control, in turn, improving overall diabetes care and giving rise to individually tailored care strategies.

Time in range (TIR)

Time in range (TIR) is the percentage or amount of time that a patient's blood glucose, as monitored by continuous glucose monitoring (CGM), remains within the target range of 70-180 mg/dL [24]. To quantify the duration a patient spends with glucose levels high, low, or in the target range, there are now five accepted, CGM-defined categories for TIR [23]. The duration in each category during the measurement period can be expressed as the number of minutes or hours per day or as a percentage of CGM glucose measurements. For example, TIR = 50% (or 12 hours/day) if half of all the CGM glucose measurements during the course of the 14 days fall within the target range. Recognizing that there may be situations where the patient or doctor would like to establish a different target TIR (e.g., 70-140 mg/dL throughout the night for patients on hybrid closed-loop therapy), the agreed-upon default TIR is 70-180 mg/dL.

Glucose variability (GV)

GV is the amount that the glucose measurement deviates from the mean or median glucose, the frequency of fluctuations, and the amplitude, or degree, of up-and-down volatility. Numerous GV measures are well-established. The majority use coefficient of variation (CV), standard deviation (SD), interquartile range (IQR), and mean amplitude of glycemic excursion (MAGE) to measure the amplitude of GV. The most persistent GV measure, CV, is not directly associated with either A1c or mean glucose. According to recent studies, a reduced or lower CV is indicated by a CV value of less than 36% and a somewhat stable glucose balance. In comparison, an unstable glucose profile is denoted by a CV value of more than 36%. The most widely used GV metric, SD, strongly correlates with both A1c and mean glucose [25-28]. In clinical studies, determining hypoglycemia is just as crucial as measuring time in range. Alternative approach for glucose management is quantifying glycemic excursions length and intensity. Trials examining the utility of CGM technologies have revealed a common theme - for people with diabetes to benefit most from the device, it must be worn almost every day [29].

Application of various AI tools for the control and management of diabetes mellitus

AI Modalities in Diabetes Management

AI modalities, such as machine learning (Random Forest, XGBoost), deep learning (CNNs), and reinforcement learning (Q-learning), analyze genetic, clinical, and lifestyle data to predict T2D risk (area under the curve {AUC} up to 0.96) and support retinopathy screening or insulin dosing [30]. These integrate with CGM and electronic health records (EHRs), processing glucose readings every 1-15 minutes for personalized care. AI identifies high-risk individuals using genetic, lifestyle, and EHR data, enabling early intervention for the Indian T2D phenotype [31].

Through the analysis of large datasets, pattern recognition, and prediction, artificial intelligence (AI) has demonstrated significant promise in the early detection and ongoing control of diabetes. AI is able to detect people who are at high risk of acquiring diabetes by scrutinizing genetic information, lifestyle factors, electronic health records (EHRs), and historical health information [32,33]. Through the analysis of data from large populations, machine learning algorithms can be trained to recognize trends that arise before diabetes develops, enabling early intervention. Since the Indian population has a distinct set of type 2 diabetes (T2D) characteristics, AI systems can be particularly beneficial in tailoring the treatment and management process. For instance, machine learning models can be taught using datasets featuring certain genetic markers and lifestyle patterns that are common among Indian patients, thus making the risk prediction and personalized treatment strategy more accurate [34].

Primarily, Random Forest and gradient boost models, such as XGBoost, have been employed to integrate genetic data (e.g., single nucleotide polymorphisms {SNPs}), lifestyle factors (e.g., diet, physical activity), and clinical factors (e.g., HbA1c, BMI) to precisely predict risk for T2D with area under the curve (AUC) values of 0.96 or more in multimodal models [35]. Such models utilize large, representative datasets, such as the Indian Diabetes Consortium datasets, to derive the complexity of T2D within the Indian population. Deep learning algorithms, such as convolutional neural networks (CNNs), have also been utilized in applications like the IDx-DR system for computerized diabetic retinopathy screening, which analyzes fundus images to identify early retinal lesions with high sensitivity and specificity [36]. In addition, reinforcement learning algorithms, such as Q-learning, have been utilized to provide personalized insulin doses for type 1 diabetic patients based on real-time CGM device measurements and patient-specific parameters, including exercise and alcohol intake [37]. These tools must be trained with substantial and diverse datasets to ensure that they are generalizable and strong, particularly in the Indian population, where there is extensive genetic and environmental heterogeneity [38]. Additionally, the development of these AI tools involves technical considerations, such as the integration of diverse data sources (e.g., genetic data, lifestyle information, EHRs) and the application of advanced algorithms like deep learning. These tools require extensive training on large, representative datasets to ensure they accurately capture the nuances of T2D in the Indian demographic. The proposed AI-based tool in the control and management of diabetes mellitus will provide functionalities discussed further.

Personalized prediction of diabetes mellitus and its stages

Maintaining target blood glucose levels in diabetes is challenging, as bolus insulin injections increase hypoglycemia risk [39]. AI-driven algorithms have emerged to analyze intricate, multidimensional records to find high-risk individuals, risk factors, and biomarkers linked with T2D, and direct tailored prevention measures. AI models analyze multidimensional data to predict T2D risk, with multimodal models achieving AUCs up to 0.96 [40]. Gut microbiome studies enhance predictions [41]. Neural networks with more single-nucleotide polymorphisms (SNPs) outperform clinical models. The majority of T2D predictive models use a single data modality, such as electronic health records (EHR); multimodal models combine metabolomic, genetic, and clinical records [32]. In one investigation, the model’s performance was improved, yielding an AUC of 0.884, which surpassed models using only genetic data (AUC of 0.876) or classical risk factors (AUC of 0.84). A similar study produced an AUC of 0.96 for the multimodal model, outperforming genomics-only (AUC of 0.586) and clinical-only (AUC of 0.798) models [42,43]. The first long-term study evaluated the gut microbiome's predictive function for T2D-related indicators when combined with traditional risk determinants and employing microbiomes in patient-curated medicine [44].

In a different study, researchers used different numbers of single-nucleotide polymorphisms (SNPs) to compare the performance of linear regression (LR) and deep neural network (DNN) models. The LR and DNN models demonstrated limited discriminative abilities when only 96 or 214 SNPs were used, and they did not outperform a clinical model based on conventional risk factors [45]. Both models outperformed the clinical-only model in terms of AUC when additional SNPs (399 and 678) were added. When both clinical parameters were combined with SNPs, the DNN models' AUC was greatly increased. These results demonstrate enhanced predictive accuracy using metabolomic, clinical, and genetic data in T2D prognosis.

Monitoring diabetes complications/adverse events and proactive interventions

Peripheral neuropathies and vascular pathologies are typical consequences of diabetes. To standardize diabetic foot snapshots, a smartphone app named "FootSnap" (Manchester, UK: Manchester Metropolitan University) was created [46]. Its stability was tested in a trial with 60 participants, using the Jaccard Similarity Index (JSI). JSI values ranging from 0.89 to 0.91 and control group values from 0.93 to 0.94 had high reliability. AI tools, such as FootSnap and thermal imaging, can detect complications [47]. CGM-based models minimize hypoglycemia false positives [48]. Random Survival Forest assesses risk and XGBoost predicts hypoglycemia. Retinal screening tools (IDx-DR, DeepDR) aid early intervention [49]. Thermal imaging technique using asymmetry analysis and a genetic algorithm was used by Kaabouch et al. to evaluate skin integrity and identify inflammation and future foot ulcers [46,50].

Insulin therapy increased the risk of hypoglycemia in T1D patients but decreased the risk of late-diabetic sequelae by reducing the average blood glucose [51]. A novel pattern classification technique using expert CGM data detected hypoglycemia episodes with minimal false positives. The utility and impact of lipid and HbA1c variability for risk assessment and survival analysis in diabetes were examined using regularized and weighted Random Survival Forest (RSF) models [52]. The results showed that due to the high risk of both diabetic complications and mortality, there was a spike in the variability of cholesterol and HbA1c values. It was also determined that inflammation had a significant role in modulating the negative effects of diabetes by relying on hypoglycemia frequency, baseline neutrophil-lymphocyte ratio (NLR), and lipid and HbA1c variability. Still, more research must be done in the future to validate the findings.

A four-year study of electronic health data applied machine learning to predict hypoglycemia. Another study employed four years of electronic health data, including laboratory and point-of-care blood glucose measurements, oxygen saturation, albumin levels, diabetes type, weight, and medications used to predict the risk of hypoglycemia. Blood glucose tests conducted throughout the hospital stay were used to supplement the model. The XGBoost model scored the best (AUC = 0.96), beating the LR model (AUC = 0.75). Globally, in terms of risk assessment for clinically significant hypoglycemia, diabetic care has incorporated AI tools, such as automatic retinal screening, which detects diabetic retinopathy (DR) from fundus images automatically. The first well-known device was the IDx-DR, which was approved by the FDA in 2018 due to its excellent clinical testing outcomes for diagnosis [53]. This tool makes DR screening easier, particularly in remote areas where patients may find it challenging to see an ophthalmologist [50]. In recent years, several highly sensitive DL algorithms have been created for DR screening, primarily aimed at detecting referable or vision-threatening DR. Nonetheless, it is crucial to recognize early-stage DR; this cannot be overlooked. Research indicates that early intervention may reverse mild non-proliferative DR and bring the condition back to a state free of disease development. To address this problem, Dai et al. created an automated and interpretable system. They verified the DeepDR system, which possesses excellent sensitivity and specificity for the initial to late-stage DR grading, retinal lesion diagnosis, and real-time image quality assessment [54].

Personalized prediction of blood glucose and treatment strategies

Diabetes-related problems decreased globally, with several approved antihyperglycemic medications for T2D [55]. Early treatment selection is crucial to prevent severe hypoglycemia, which can cause cardiovascular complications [56]. Machine learning-aided clinical practice can identify patterns for better decision-making [57,58].

Continuous exogenous insulin therapy is necessary for T1DM patients to control blood glucose levels and avert the disease's long-term consequences. A study was conducted to create and evaluate a general reinforcement learning (RL) framework for using clinical data in the individualized treatment of type 1 diabetes [59,60]. Machine learning optimizes antihyperglycemic therapy to reduce complications. Q-learning tailors insulin doses for T1D using CGM data, requiring further validation [61,62]. This work introduces Q-learning, a model-free data-driven RL algorithm for suggesting insulin dosages to manage blood glucose levels in T1DM patients according to the patient's status as reflected by body mass index, level of physical activity, glycated hemoglobin (HbA1c) levels, and alcohol consumption. This pilot work demonstrates that tailored insulin dosage suggestions for T1DM patients are possible through an RL algorithm to achieve proper glycemic control. Nevertheless, further research involving a larger sample size is needed.

The estimation of insulin injection timing in T1DM patients is aided by the evaluation and modelling of glucose fluctuations. To achieve this, the Tesseratus hybrid model was developed to predict glucose oscillations for up to 4 hours during the day and 8 hours at night [63]. Compartment models and data-driven methods, such as machine learning, are the foundation of this concept. Reactive agents collect data, monitor errors and ordinary differential equation (ODE) variables, and transfer this information to intelligent agents, such as recommender, predictor, and machine learning (ML) models, to forecast glucose oscillations. Tesseratus was proposed as a model for classifying a glucose prediction model that reduces the risk of long-term complications for individuals with type 1 diabetes. Insulin recommendations are provided by logic-based AI systems. Using case-based reasoning (CBR) and an intravenous insulin bolus calculator, researchers have attempted to ascertain how eating, drinking, and exercise schedules affect a person's glucose metabolism and reach target glucose levels [64].

The FDA has approved the smartphone software program known as the WellDoc diabetes management system (Columbia, MD: WellDoc, Inc.) in the United States, and most medical insurance plans cover it. Through real-time online interaction, clinicians can utilize this system with patients' blood sugar records to offer personalized suggestions and feedback. Patients can enter information about their diets and medications and track their blood sugar levels in real-time by using the app that physicians can recommend. A randomized control trial, which included patients with type 2 diabetes, was conducted by Quinn et al. utilizing this app [65]. The results showed that patients who used the smartphone app to manage their diabetes had A1c readings that were 1.2% lower than those of the control group. In a study, the effectiveness of the TangTangQuan (TTQ) (Shenzhen, China: Shenzhen Aibaowei Biotechnology Co., Ltd.) mobile health app was assessed in treating type 1 diabetes in China. Patients used the TTQ system to record and upload their diabetic diaries [66].

As a patient's condition worsens, treatment escalation associated with diabetes is another crucial component of the medication therapy regimen. Several studies have attempted to help doctors by identifying the best times to accelerate a patient's care. Murphree et al. used several AI techniques to determine and forecast T2D patients for whom metformin monotherapy is likely to be unsuccessful and necessitate medication escalation [67]. For training their models, they utilized demographic, comorbidity, and HbA1c datasets. Similarly, Fiorini et al. used medical visit information to train an artificial neural network to forecast when patients might be required to switch from metformin monotherapy [68]. In a study, an EHR-integrated approach was implemented for forecasting variations in HbA1c linked to other T2D options for treatment, exhibiting the potential of AI-based tools in clinical practice [69].

Discussion

Persistent differences in diabetes outcomes, which can be caused by a complex interaction of biological, socioeconomic, healthcare, and behavioral variables, are a major public health problem. To provide individualized therapy methods for people with diabetes and the need for measures to address these gaps, it is imperative to comprehend these reasons. Socioeconomic issues, such as wealth disparity and educational attainment, are crucial because they affect people's access to safe spaces for physical activity, wholesome food, and high-quality healthcare [70,71]. In the Indian context, traditional Ayurvedic approaches, which emphasize holistic lifestyle and dietary interventions, can complement AI-driven precision medicine by addressing cultural preferences in diabetes management [72]. A lack of knowledge about managing diabetes is linked to lower educational attainment, and this can lead to inadequate medication adherence and poor self-care habits. Diabetes management may also be impacted by cultural variations in food practices, perspectives on sickness, and attitudes toward medical care. Language problems may also prevent certain populations from efficiently communicating with healthcare practitioners. Certain ethnic groups, including Native Americans, African Americans, and Hispanics, are more susceptible to diabetes and associated consequences because of environmental signals [70,73,74]. Comorbidities, which are more common in certain racial and ethnic groups, or the presence of other medical conditions, such as obesity or hypertension, can make managing diabetes more difficult and result in harm. Expanding new policies, such as Medicaid expansion and health insurance subsidies, can assist more people in obtaining regular personal diabetic treatment, therefore increasing access to affordable healthcare. Innovative methods, such as telemedicine and its offerings, can enhance the availability of diabetes treatment, particularly in remote or underprivileged regions, by enabling patients to obtain direction and supervision without requiring regular in-person consultations [75]. Several community-based initiatives that incorporate the cultural norms and values of certain communities can enhance the efficacy and involvement of diabetes treatment. To guarantee patient understanding and close communication gaps, instructions and translation services should be in numerous languages. To address socioeconomic determinants of health, we can set up several food security initiatives. Programs like farmers' markets, community gardens, and food subsidies that provide access to healthful foods can support diabetics in eating a balanced diet.

Explainable AI (XAI) improves transparency in AI-powered tools, which is essential for creating trust among patients and clinicians in diabetes management. XAI techniques, such as Local Interpretable Model-Agnostic Explanation (LIME) and Shapley Additive Explanation (SHAP), offer explainable knowledge of AI predictions, allowing clinicians to authenticate glucose predictions and treatment suggestions. This is especially important in high-risk environments, such as diabetes management, where mistakes can result in dire consequences, such as hypoglycemia [76]. Ethical and regulatory challenges, including FDA and cross-entropy (CE) approvals, are significant hurdles for AI adoption. The FDA’s Software as a Medical Device (SaMD) framework requires fairness audits to mitigate biases, ensuring equitable performance across diverse populations, such as the Indian T2D phenotype [77].

MHealth apps, including WellDoc and Tidepool (TTQ) (Palo Alto, CA: Tidepool), combine CGM to offer real-time glucose tracking and customized insulin dosing advice. A 2023 meta-analysis showed that mHealth apps reduced HbA1c by 0.5-1.2% in T2D patients, with WellDoc demonstrating impressive efficacy in self-management (OR: 1.8, 95% CI: 1.3-2.5) [78]. However, effectiveness in low-resource settings is limited by digital literacy and infrastructure deficits, highlighting the need for accessible app designs. These advancements, combined with XAI, can enhance trust and equity in AI-driven diabetes care, particularly for the Indian population [79].

Low socioeconomic status (SES) can exacerbate the challenges of managing type 1 diabetes (T1D) and act as an independent risk factor for chronic and acute diabetes complications. Environmental factors contributing to low SES and T1D need to be addressed more to describe the association between SES factors like education, income, employment, and insurance coverage with T1D outcomes in both acute and chronic complications and comorbid cases [80]. However, among T1D patients who maintain adequate chronic control, the risk of complications and even mortality is connected with a person's SES. The causes of these variations in glycemic control in patients with T1D, regardless of their varying socioeconomic statuses, remain unknown. Several factors may be responsible for these differences, including diet, such as access to fresh fruit and vegetables versus fast food, physical exercise, including the availability of or access to spaces for physical exercise or the practice itself, health literacy, or regular visits to an endocrinologist [81,82]. Regardless of an individual's socioeconomic status, new technologies can improve glycemic control and lower the risk of diabetes-related microvascular chronic complications [83].

Self-management technologies are essential for preventing T1D complications and achieving ideal glycemic control [84]. However, due to cost, infrastructure, and accessibility issues, genetic factors are not easily accessible in many countries, which limits the applicability of artificial intelligence (AI) tools that rely on genetic variables, especially in diseases where medical treatment and patient behavior significantly influence disease outcomes. Gene therapy is even prohibited in most countries [85]. Thus, special attention must be given towards managing long-term illnesses like diabetes or hypertension through effective medicinal interventions and ensuring patient adherence to treatment protocols. These aspects are equally critical for achieving optimal diabetes management. In these situations, AI models that examine these critical non-genetic components while prioritizing genetic elements can be created, providing actionable insights into the diagnosis and treatment of diabetes. Further, improvements in delivery techniques and CRISPR/Cas9-based gene therapy for diabetes are propelling this field forward [86]. To overcome the current obstacles and guarantee the security and effectiveness of these treatments, more research is anticipated.

The use of CGM in our study is susceptible to false signals affecting its reliability and user satisfaction. Frequent alerts may cause desensitization, leading users to ignore or disable notifications. Certain drugs, such as acetaminophen, can chemically interfere with sensors, producing unreliable readings [87]. Survey data indicate that 41% of users discontinued CGM within a year, due to discomfort or device malfunctions [88]. Dialysis patients or individuals eligible for Medicare at the age of 65 years may lose CGM coverage. Precision medicine addresses limitations mentioned through advanced decision-support systems, funding for educational initiatives, and iterative device upgrades [89,90].

Current evidence points to the emerging role of AI in the management of T2D, including offering personalized care to heterogeneous groups, like the Indian phenotype. Screening for diabetic retinopathy using AI has also been shown to be cost-effective and improve early detection, which is important in Indian populations with high type 2 diabetes prevalence [91]. These advances highlight the ability of AI to provide individualized interventions, treating the unique insulin resistance and adiposity issues of the Indian T2D phenotype. The lack of population heterogeneity in AI model training impacts the generalizability. The influence of socioeconomic status (SES) on diabetes care is examined, but it does not address to any extent how AI tools can be modified to treat these disparities. Subsequent studies should aim to develop inclusive AI models that synthesize genetic, environmental, and lifestyle variations, thereby preventing algorithmic bias and ensuring fair diabetes care.

Challenges and future directions

The implementation of AI-driven precision medicine in T2D is hindered by generalizability challenges, as models often overlook the heterogeneity of the population in terms of genetic and socioeconomic determinants, which risks biased outcomes [92]. Algorithmic bias poses a threat to amplify health inequalities, especially in diabetes management, where underrepresented populations, such as ethnic minorities or low-income groups, can result in biased predictions [93]. Bias emerges from incomplete data that do not include cultural, linguistic, genetic, or environmental differences in underserved populations, and this amplifies disparities in low- and middle-income countries [94]. Thus, multiple datasets and fair-aware algorithms are necessary, along with open science methodologies to counteract bias in the form of transparent data sharing and diverse model training [95]. Data privacy takes precedence in the face of evolving regulations, which require safe storage and encryption to protect sensitive health information. This is particularly important for AI systems managing diabetes, as they often handle large patient datasets that are susceptible to breaches [96]. Further, the integration of AI in India's healthcare system is being hindered by limited digital infrastructure and expensive solutions, particularly in rural regions, where access to high-quality data and computing resources remains uneven [97]. Additional challenges include device interoperability, ethical considerations in algorithmic decision-making, and the need for adaptable regulatory frameworks to ensure the safe deployment of these technologies [98]. Federated learning can promote generalizability and privacy by having models learn from decentralized data without sharing data centrally. This facilitates collaborative development of AI across institutions while maintaining the confidentiality of patients with diabetes [99]. Explainable AI can advance trust by making predictions transparent, allowing clinicians to comprehend model choices in real-time, such as during glucose monitoring or complication prediction. These methods should be confirmed in the future in India to enable equitable, scalable diabetes management, with an emphasis on longitudinal research, integration with patient-oriented systems, and the remediation of disparities through personalized, AI-based interventions.

## Conclusions

In this study, we have investigated the use of AI for managing diabetes, with a special emphasis on incorporating CGM data and the distinctive phenotypic features of T2D in the Indian population. By using big data, AI instruments can identify high-risk subjects for developing diabetes, who can then be treated early and develop personalized treatment plans that incorporate genetic, lifestyle, and environmental determinants. Consequently, most AI-driven tools and technologies that support personalized medicine are focused on the diagnosis, prognosis, and treatment of individuals. Moreover, by addressing these challenges, AI holds the potential to revolutionize diabetes management, offering more effective and personalized care and ultimately improving patient outcomes on a global scale.
